# Supplementary material for: Uncovering miRNA-mRNA Regulatory Modules in Developing Xylem of Pinus massoniana via Small RNA and Degradome Sequencing
Source: Int J Mol Sci. 2021 Sep 21;22(18):10154. doi: 10.3390/ijms221810154 (PMC8472836; doi:10.3390/ijms221810154)
Supplement: Supplementary file 1 [file ijms-22-10154-s001.zip › ijms-1365557-supplementary/Additional file S2.zip!/Additional file 2/novel_103_Cluster-18410.26812_408_TPlot.pdf]

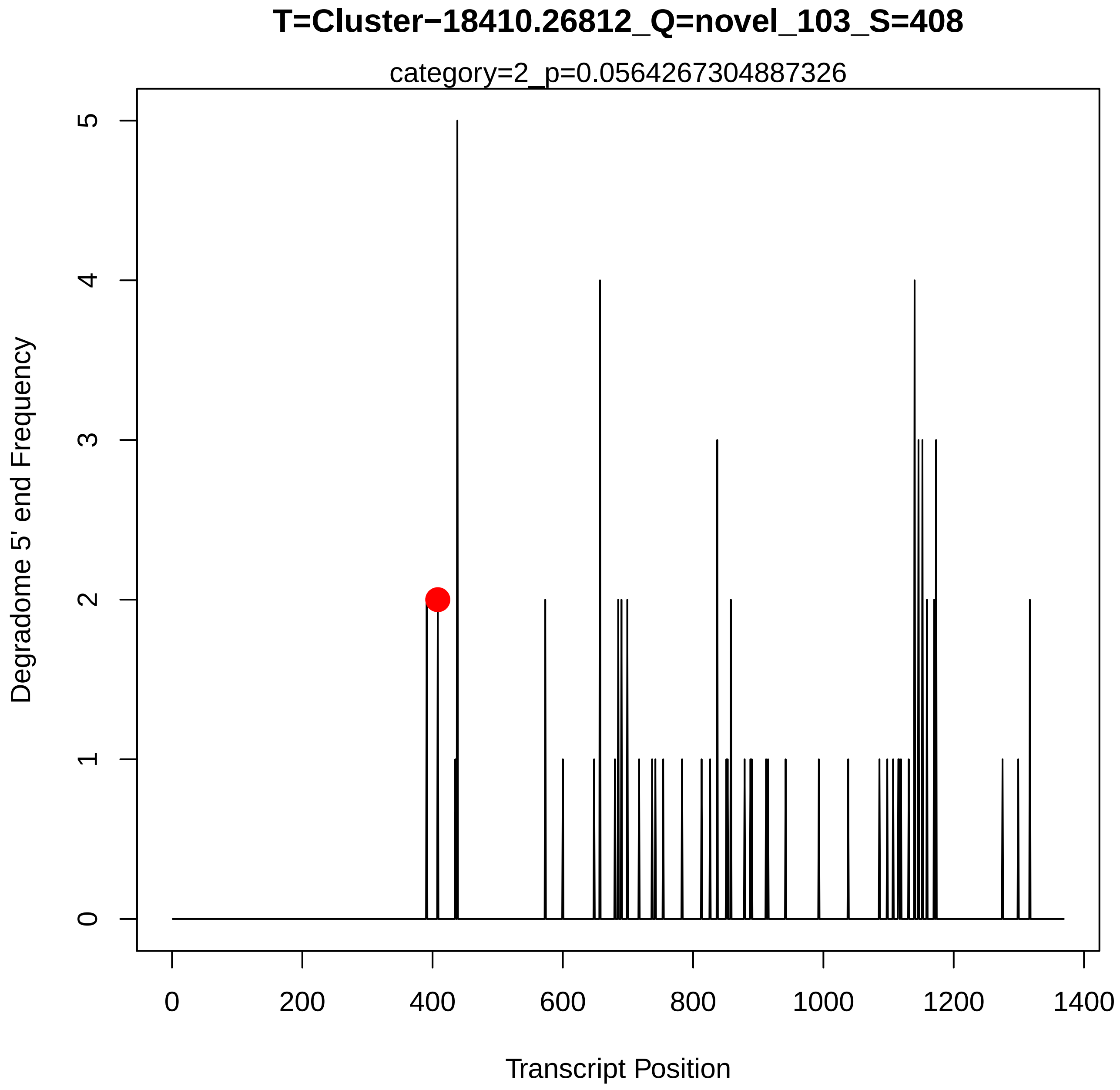

T=Cluster−18410.26812_Q=novel_103_S=408
category=2_p=0.0564267304887326
Degradome 5' end Frequency
0
1
2
3
4
5
0
200
400
600
800
1000
1200
1400
Transcript Position
